# The New Poor-Law Orders: Their Substance and Effect on Infirmary Work

**Published:** 1914-01-10

**Authors:** 


### THE NEW POOR-LAW ORDERS.

### Their Substance and Effect on Infirmary Work.

In The Hospital of August 23 and 30 and of September 27 last year the report of the Departmental Committee appointed to examine the various Poor-Law Orders with a view to their consolidation, with such amendments as might be considered necessary, into one or more new Orders, was considered and criticised. Two new Orders founded upon this report have now been issued and are to come into force on March 31 of this year. The first Order—the Poor-Law Institutions Order (1913)—concerns itself with the general administration of the workhouse; the second and much shorter Order—the Poor-Law Institutions (Nursing) Order (1913)—with the provision, qualifications, and tenure of office of the nursing staff of workhouses. In the present article the most important of the provisions of these Orders from the medical officer's point of view will be enumerated, and in a subsequent article these provisions will be commented upon and criticised.

The two Orders are founded to a very large extent upon the recommendations of the Departmental Committee, but the criticisms of The Hospital and other papers evoked by the publication of the report of that Committee have led to several important and valuable modifications. The wording of the new Orders is in many instances much clearer and less ambiguous than that of the Draft Order prepared by the Committee. It is made quite clear, for instance, that they are not to apply to separately administered infirmaries or to separate institutions provided wholly for children.

The first striking change from the existing order of things concerns the children. After March 31, 1915, children over three years of age are not to be kept in the general mixed workhouse for a longer period than six weeks unless they are sick. This is a reform which has long been demanded. Most Boards of Guardians have indeed already provided accommodation outside the workhouse for the healthy children in their charge, but the fact that on January 1, 1913, there were still more than 8,000 children over three years of age in the ordinary wards of workhouses shows the necessity for the central authority to insist upon the backward Boards falling into line with the more progressive. A change which will very directly affect medical officers is that which requires a record paper to be kept in the case of every inmate of a sick ward just as is done in most separately administered infirmaries and in the voluntary hospitals. This is already done in some workhouses, but in the majority of cases the only records that have been kept hitherto are the scanty details required in the workhouse medical relief book—practically a dietary book with a column for diagnosis. This cumbersome and almost useless book is to be abolished, and its disappearance will be hailed with joy, but the keeping of a detailed record of the condition on admission, the progress and treatment of each case as required by the new Orders, will mean a very great addition to the work of most medical officers. Their work is to be increased in many other ways. They must keep a record of the condition on admission and the fitness for work of every inmate, examine every infant under eighteen months at least once a fortnight, and every child over that age at least once a month and keep a record of their examinations, and give to the medical officer of health any information the Local Government Board may direct with respect to cases of sickness or death within the institution, and to the clerk to the Guardians any information on matters affecting their office required by him for the purpose of any report or for the business of the Union. They are still required, as at present, to report to the Local Government Board every sudden or accidental death occurring among the inmates of the institution, but it is definitely stated that they are not required to report deaths from accidents occurring at a time when the deceased was not in receipt of relief, and they are no longer required to report to the Local Government Board outbreaks of infectious disease in the workhouse.

Every Board of Guardians is required to appoint a house committee. This committee will not be an executive body, but will act under the directions of the Guardians and be subordinate to them. Nevertheless many important duties are prescribed for this committee. It is to cause the institution to be inspected and the stores examined at least once a fortnight; to arrange for surprise inspections; to consider at each meeting the reports of the master, the medical officer, the matron, and superintendent nurse; to interview all inmates admitted since the previous meeting; to investigate complaints by inmates and to afford them an opportunity of making complaints and applications; and to report to the Guardians periodically upon the condition of the institution and the suitability and sufficiency of the accommodation provided for the various classes of inmates.

The Guardians are also given the power to appoint a committee of women, whether Guardians or not, to visit the women, children, and infants, and the wards in which those inmates are maintained. The duties of this committee are to be such as the Guardians may from time to time prescribe. The powers to be exercised by these committees are much more clearly defined than was the case in the Draft Order, and it is definitely laid down that they cannot demand the production of documents or call for the attendance of officials. Additional powers are given to the Guardians. They are required to make regulations (1) for the searching of inmates and the prohibition and disposal of articles not proper to be brought into the institution; (2) for the hours and places of meals and work, and the hours of rising and going to bed; and (3) for the bathing and cleansing of inmates other than infants or those in the sick wards or lunatic wards. All such regulations must be submitted to the Local Government Board, which retains the right to disallow, modify, or suspend them.

The medical officer is required to draw up a code of instructions for the bathing and cleansing of the inmates of the sick wards and lunatic wards and the infants, and to give any directions he may think necessary in regard to the bathing and cleansing of any particular inmate. The rights of inmates are more carefully guarded under the new Orders than under the Draft Order. One point about which some doubt was felt is cleared up by the circular-letter accompanying the Orders. It is there stated definitely that the circular-letters and minutes of the Local Government Board and their predecessors are in no way superseded, except in so far as some express provision on the matters dealt with by the circulars or minutes is contained in the new Orders. Although it is contemplated that inmates making complaints will be brought before the House Committee as a rule, such inmates are to be given the option of appearing before the whole Board if they so desire. The effect of the criticisms to which the Draft Order was subjected is seen in the appearance of the new order of clauses: (*a*) ordering the restitution to the inmate when discharged of the clothes worn by him and the articles found upon him when admitted, or the provision by the Guardians of suitable and sufficient clothing if the inmate's own clothes are not restored; (*b*) providing for the access of the father and mother of an infant or child in the same institution at least daily to the infant or child; (*c*) forbidding the employment of an inmate on any work which in the opinion of the medical officer would be injurious to his health; (*d*) making it the duty of the clerk to report forthwith to the Local Government Board any instance in which the number of inmates fixed by the Board in any ward or institution is exceeded; (*e*) forbidding the punishment of any inmate pronounced by the medical officer to be pregnant, unless the medical officer shall have previously certified in writing that no injury to the inmate's health is reasonably to be apprehended from the proposed punishment; (*f*) forbidding two inmates, either of whom is over seven years of age, to occupy the same bed except in the case of a married couple allowed to live together and (*g*) ordering the master to notify any married couple who have both reached the age of sixty that they have the right to live together.

With regard to the nursing arrangements in the workhouse, the provisions of the Draft Order have been adopted with some slight modifications. A superintendent nurse is to be appointed in every workhouse with more than 100 beds for sick inmates. She is in future to perform in the sick wards the duties which would otherwise be performed by the matron, and is to make reports to the House Committee on matters affecting her duties. Where the institution possesses less than 100 beds for sick inmates but has three or more nurses, a head-nurse is to be appointed; and if the matron does not possess the nursing qualifications required of a head-nurse the latter officer is, in matters relating to the treatment and nursing of the sick, to act immediately under the directions of the medical officer. Both the superintendent nurse and the head-nurse, unless she is holding the office of superintendent nurse at the date of the operation of the Order, must hold a certificate of three years' training from a training school for nurses maintaining a resident medical officer, and must also be a midwife certified under the Midwives Act, 1902.

